# Mothers’ experiences of quality of care and potential benefits of implementing the WHO safe childbirth checklist: a case study of Aceh Indonesia

**DOI:** 10.1186/s12884-019-2625-8

**Published:** 2019-12-03

**Authors:** Siobhan Doria, Farah Diba, Suryane S. Susanti, Sebastian Vollmer, Ida G. Monfared

**Affiliations:** 1 Centre for Modern Indian Studies & Department of Economics, University of Goettingen, Göttingen, Germany; 2 Universitas Syiah Kuala, Banda Aceh, Indonesia

**Keywords:** Safe childbirth checklist, Maternal health, Maternal rights, Aceh, Indonesia

## Abstract

**Background:**

In an effort to mitigate missed opportunities to provide high-quality care, the World Health Organization (WHO) has developed the Safe Childbirth Checklist (SCC) to support health providers perform essential tasks. Our qualitative study is a baseline assessment of quality of care (QoC) perceived by mothers who gave birth at health facilities aiming to highlight areas where implementing the SCC can potentially improve the QoC as well as areas that are not part of the SCC yet require improvement.

**Methods:**

Assessing the overall experience of care, our qualitative study focuses on 8 out of 29 items in the checklist that are related to the personal interactions between healthcare provider and mothers. Using a set of semi-structured questions, we interviewed 26 new mothers who gave institutional births in Aceh province in Indonesia.

**Results:**

Our findings revealed some gaps where implementing the SCC can potentially improve safety and QoC. They include communicating danger signs at critical points during birth and after discharge, encouraging breastfeeding, and providing mothers with information on family planning. Moreover, taking a qualitative approach allowed us to identify additional aspects such as need for clarity at the point of admission, maintaining dignity, and protecting mothers’ rights in the decision-making process to be also essential for better QoC.

**Conclusions:**

Our study highlights the need to actively listen to and engage with the experiences of women in the adaptation and implementation of the checklist. While our findings indicate that implementing the SCC has the potential to improve the quality of maternal care and overall birth experience, a more holistic understanding of the lived experiences of women and the dynamics of their interactions with health facilities, care providers, and their birth companions can complement the implementation of the checklist.

## Background

The global commitment to improve maternal health by reducing maternal mortality has largely focused on addressing the direct causes of pregnancy-related death. With maternal death as the measurable outcome of progress and success, interventions have often reduced maternal mortality to a medical problem, where lack of access to skilled biomedical providers has dominated the agenda for making pregnancy safer [1, 2]. In an effort to support health workers perform essential tasks and improve quality of care (QoC) for mothers and newborns during childbirth, the World Health Organization (WHO) has developed the Safe Childbirth Checklist (SCC). The 29 item checklist (Additional file 1) targets 4 critical pause points in clinical care: on admission of the mother to the birth facility; just before delivery or caesarean delivery; soon after birth (within 1 h); and before discharge [3–5]. The checklist provides an organized list of evidence-based essential birth practices targeting the major causes of maternal deaths globally.

The use of checklists in health care is becoming increasingly common to manage the complexities in clinical care and improve communication during clinical practice. Across a wide range of resource settings, the literature on health checklists shows their ability to reduce risks by standardizing and improving the translation of information between providers, ensuring a consistent standard of care and decreasing human error under stressful conditions [6, 7]. While some evidence suggests that implementing the SCC can save newborn lives [8] and improve QoC when appropriately adapted [9], the implementation of the checklist has yet to correlate with significant improvements in maternal and neonatal mortality and patient care [10–13].

Although the WHO recommends adapting and modifying the checklist to fit the local context [5], so far such efforts have not included the voices of local women interacting with the health facilities. With the growing recognition of the need for respectful maternity care and attention to women’s individual, cultural, personal, and medical needs, it is critical to thoughtfully engage with the experiences of women who are in the position to provide deep information about the realities that they face [14, 15]. The SCC preceded the development of the WHO’s framework [16] and standards for improving quality of maternal and newborn care in health facilities. This framework includes both provisions of and experiences of care that aims to achieve the coverage of key practices in addition to people-centered outcomes, recognizing that poor quality of care contributes to morbidity and mortality. The framework contains 8 domains of quality of care to assist in translating it into practice, and it is accompanied by statements to aid practitioners in delivering measurable outcomes [2]. Emphasizing QoC as “the extent to which health care services provided to individuals and patient populations improve desired health outcomes”, the WHO calls for care that is safe, effective, timely, efficient, equitable, and people-centred [2]. Integrating elements of the framework of QoC into the SCC can play an important role in improving the birth experiences of mothers by promoting supportive and effective communication throughout the birth [17–19]. The flexibility of the checklist allows for a design tailored to local needs which can ensure that minimum requirements for a safe and satisfactory childbirth experience are met [9]. Moreover, the structured order of the SCC and the availability of the implementation guidance can help healthcare providers to use it as a tool to assess and improve QoC [5].

Indonesia has one of the highest maternal mortality rates (MMR) in Southeast Asia with 192 maternal deaths per 100,000 live births in 2015 [20]. Despite considerable efforts to meet the 5th Millennium Development Goal (MDG5), Indonesia missed to meet its target of 102 [21]. It also should be noted that the 2015 national report of population statistics indicate a much higher figure for MMR, 305 per 100,000 live births [22]. This means that in order to meet the Sustainable Development Goal 3 (SDG3) target of 70/100,000 by 2030 [23], an annual reduction rate of 9.5% is needed [24]. Wide regional disparities exist in Indonesia, with extensive variations in MMR by province. Aceh, the setting of our study, is an autonomous province and is estimated to have an MMR of 134 per 100,000 live births and a neonatal mortality rate of 28 per 1000 live births [25, 26]. Data from the Indonesian Ministry of Health (2018) indicates that 79% of antenatal care and 65% of births in Aceh are performed by skilled birth attendants. Furthermore, 77% of births were normal deliveries; there were 29% of births took place in private clinics and 15% in public hospitals [27]. Additional file 2: Table S1 and Additional file 2: Table S2 provide further details about some of the key maternal care indicators in Aceh [28, 29].

The present qualitative study aims to translate the perception of QoC from Acehnese women who gave institutional birth in this province. Using a qualitative approach that enables capturing an in-depth insight into women’s experiences, we aim to highlight key areas of importance for mothers. Their insights can not only help to improve QoC, but also potentially highlight new recommendations for the SCC.

## Methods

The qualitative study reported in this article is part of a larger randomized control trial (RCT) and was carried out prior to the implementation of the SCC in Aceh province. The RCT sampled 32 of 40 eligible health facilities in three districts of Aceh Province: the capital city of Banda Aceh (12 facilities) and the regencies of Aceh Besar [7] and Bireuen [13]. These facilities are official basic emergency obstetric and newborn care (BEmONC) providers, and include community health centers (Puskesmas), private midwife clinics, and private and public hospitals. All of the private and public hospitals provided comprehensive emergency obstetric and newborn care (CEmONC). Only 33% of the private midwife clinics and none of the Puskesmas that participated in this RCT provided this service. During the assessment phase (August to October 2016), quantitative indicators about the individual health facilities were collected and experts in childbirth and maternal care in Aceh were consulted about the adaptations of the checklist. The qualitative study was conducted during the assessment phase and thus did not influence the design of the RCT, as the SCC was implemented in 12 treatment facilities from October 2016 to April 2017. Adopting a concurrent design created the space to gain an in-depth understanding of new mothers’ birth experiences and their perceptions of QoC with a specific emphasis on the points highlighted in the SCC. Of the 29 items in the checklist, we focused on 8 items that require effective communication and collaboration between the mother, her family, and healthcare providers.

Empirical research was conducted between August and October 2016, utilizing a combination of convenience and snowball sampling. With considerations for cultural sensitivity, we did not find alternative methods of sampling (outside of the women-carer network) to be appropriate. Eligibility for inclusion in the study was based on the following criteria: gave birth in one of the study health facilities within the last 6 months; resides in one of the three study districts; availability and willingness to participate; and the ability and capacity to consent. Mothers were approached directly at the health facilities and were given a brief introduction to our research. Contact information was exchanged with those who expressed interest in participating, and interview times were arranged in follow-up phone calls. Upon completion of the interview, the mothers often recommended additional new candidates who fit the eligibility criteria of the study.

A semi-structured interview guide was developed, pilot tested with 3 mothers, and adjusted for clarity and flow of questions (Additional file 3). Some interviews were done in Bahasa Indonesia and Acehnese language and were approximately 1 h in length. The interviews were conducted by two trained qualitative health researchers, one from the University of Göttingen and the other from Syiah Kuala University, with two local Syiah Kuala University students acting as translators. Both of our qualitative team leaders have Masters in Public Health and are trained in qualitative and mixed method research. All participants signed written consent forms which were reviewed verbally prior to the interview. Interviews were audio recorded for the purpose of transcription and analysis. To minimize any potential harm to the mothers, pseudonyms have been used and other identifying information such as the names of the health facility and staff have been anonymized in the analysis and reporting of the study results. The interviews occurred in the homes of the mothers and were often conducted in the presence of family members such as husband and/or mother of the interviewee. We stopped conducting the interviews when data saturation was reached and no new information or themes emerged.

The data collection and analysis were done concurrently using an iterative process for the purpose of better developing a rapport with each interview, thus ensuring trustworthiness in our findings. Interviews were transcribed verbatim, translated into English and analysed using a six-phase inductive thematic analysis [30]. The analysis began by a thorough reading of the transcripts before we developed the code structure. A hybrid approach (top-down/deductive, searching for themes driven by theory and bottom-up/inductive, searching for themes driven by data) was employed whereby two researchers independently analysed and coded the interviews. Coding reliability was established between the two coders as line-by-line coding was performed independently before the researchers compared results. Further analysis of the connections between the codes and their themes was conducted using Nvivo 12 Plus.

This study holds ethical approval from the ethics committee at the University of Göttingen, Germany, as well as the ethics committee at Syiah Kuala University in Banda Aceh, Indonesia.

## Results

Of the 26 interviews, 11, 7, and 8 where carried out in three different districts (names removed to maintain confidentiality). The details of background characteristics of participants are given in Table 1. The mothers who were interviewed were 21 to 46 (average 30) years of age, gave birth between 3 days and 6 months prior to the interview, and had between 1 and 6 children.

**Table 1:** General characteristics of mothers interviewed (*N* = 26)

Characteristics	Count
Age	
20–25 years	4
26–30 years	12
31–35 years	5
Above 35 years	5
Education	
Completed primary school	5
Completed senior high school	11
Completed bachelors	7
Above bachelors	3
Occupation	
Stay at home mother (SAHM)/housewife	19
Teacher	4
Farmer	1
Other	2
Neonatal mortality	2
Type of delivery	
Normal	14
Caesarean-section	12

There were two cases of neonatal deaths that occurred within 2 days of giving birth. Additional file 2: Table S3 presents the characteristics of the healthcare facilities where the participant mothers gave birth. Table 2 presents the count and ratio of mothers’ experiences with the checklist practices. These figures indicate that there were noticeable missed opportunities to provide quality care to women.

**Table 2:** Ratio of mothers perceived care for steps indicated in the SCC prior to its implementation (*N* = 26)

Checklist item on WHO SCC	Count (%)
Birth companion was present during birth	24 (92.3)
Call for help during labour was encouraged	10 (38.5)
Call for help soon after birth was encouraged	10 (38.5)
Started breastfeeding and skin-to-skin contact	18 (85.7*)
Stayed at the facility for at least 24 h	14 (53.8)
Family planning was discussed and offered before discharge	8 (30.8)
Follow-up care was arranged before discharge	12 (46.2)
Danger signs before discharge were explained	8 (30.8)

*The ratio is adjusted excluding those cases where mother/baby conditions were complicated

The presence of a birth companion is encouraged to provide support to the mother during labour, childbirth, and in the post-partum period. When appropriate, birth companions can be used as assistants to address staff shortages and inconsistent attendance. While most of the mothers interviewed had a companion present, this did not always translate to good care. For example, some felt helpless and overwhelmed when too many people were present: “*felt uncomfortable because there are too many people with me when I was about to give birth, but I felt too ashamed to ask them to leave*”. In some instances, interactions between mother and birth companion hindered the ability of women to express their desires: *“Actually, the one who should sign [the consent form for caesarean section] was my husband... but he wanted me to give birth by normal delivery. But then I couldn’t stand it [the pains] any longer ... I called my father to come here and he came [and signed the consent form]. That was what I wanted. and asked [my husband] several times... I felt really frustrated”.* While the checklist encourages a birth companion to be present at birth, simply adding this point dismisses the deeply entrenched culture and identity of Acehnese women [31] [32]. Exploring the relationship between the mother and her birth companion, the attitudes of providers towards the role of companions in birth decisions, and the institutional support for the presence of birth companions, can provide insight into barriers to QoC and ensure that the companion benefits the mother.

In additional to a birth companion, mothers required more information to meaningfully shape their birth experience. Although all the mothers we interviewed confirmed that they received monitoring during labour, they frequently stated that they were given little to no information about their care and were not consulted or spoken to by the doctors unless they demanded information. Thus, mothers and their companions were unaware of when to call for help during labour. Of the 16 mothers who said that they did not receive any clear guidance, 10 gave birth normally and 6 had caesareans. While it is possible that staff were monitoring the mothers as they prepared for C-sections, the participants often spoke of not being informed about procedures before they happened or receiving the results: *“People don’t ask, they don’t tell, they take your blood and say see you”*; *“in the room there is no button to call a nurse for help”; “they didn’t respond fast when we asked them to check if the IV is full”*; *“I don’t want them to go out. What if an accident happens and no one is here”; “we [had to] asked them to check [on me] … sometimes we had to wake them up; some others [patients] said don’t call that nurse (she’s sensitive), just asked the other one”*. Mothers who gave birth normally felt that they were less informed, particularly compared to those who gave birth at a local community centre (Puskesmas). The lack of information led mothers to feel that decisions were being made *for* them rather than *with* them. Particularly concerning was the fear expressed by mothers and their families if they demanded information and explanations: *“When I disagreed with the staff about a C-section, they told me I could sign myself out and go home. I didn’t like that … they said, if you don’t want to have operation the doctor would not be responsible for your decisions.”*

Similar themes were expressed by almost half the mothers regarding **early initiation of breast-feeding.** Aside from 5 cases in which there were complications (2 cases of newborn deaths and 3 cases of C-section births where the mothers’ condition was found to be unsuitable for early breastfeeding initiation), many mothers indicated that the healthcare providers were not patient enough to explain and encourage breastfeeding. For example, one mother said that upon seeking assistance with breastfeeding, she was told that it was not important. Staying at the facility for 24 h after giving birth did not take place in most of the cases with normal births as it was perceived unnecessary. In particular, those mothers who were accompanied by their family members staying at the facility were mindful of being a burden on others: “*it was not because I was not comfortable [at the facility], but because I felt sorry for her [the companion]”*.

Family planning presented particular complexities, as a large number of women did not receive information from their care providers. While most women stated a desire to learn more about family planning and their available options, they often turned to their family, friends, social media, or mosque for information. In 3 cases, during the interviews, mothers were informed by their husbands that intrauterine birth-control (IUD) devices had been implanted without the mother’s knowledge or consent. In some instances, men act as ‘gatekeepers’ to women’s access and utilization of family planning, with husbands making decisions about women’s contraceptive use: “*I heard about that, but I don’t have any idea if they really asked my husband to sign it because I didn’t ask him … the staff just said they installed the IUD”*; *“this new intern said I am going to install the IUD and this is the [right] time for you to get it. But I don’t know why they install it on me because I don’t know [much about it] and I didn’t discuss it with him [my husband]. When he came to the room, I asked him and he said he didn’t even know about it.”* Indeed, throughout the interviews it became clear that the men felt overwhelmed in the facility and felt pressured to sign multiple forms that they often admitted not reading. However, in some cases consent from the husband proved to be an impediment to women’s choice and a barrier to contraceptive use. The desire to have male children often dominated the conversation and this opposition to contraceptives by men was a key reason why women were not using contraceptives.

Prior to leaving the hospital, only half of the mothers were informed about follow-up care: *“Before I was discharged from the hospital, the doctor said that I need to follow up my condition at the doctor’s private clinic.”* However, many of the women who had normal births did not receive clear guidance on the necessity of follow-up care and how to access it if needed: *“Especially people who are being hospitalized and give birth normally, they don’t ask questions; they give birth and are sent away. They [staff] say, go home.”* Similarly, only in very few cases new mothers and their family were informed about the danger signs to look out for after discharge. Not receiving guidance on danger signs was particularly common for mothers with uncomplicated births. However, there were also examples of parents who were informed about the danger signs and the importance of follow-up care, but chose not to follow the instructions: *“the doctor gave me advice to go to see the paediatrician … but the people around me kept saying it’s just normal … the midwife said if I want to go to see the paediatrician, they will refer me to one. But my husband and I decided not to see the paediatrician”.*

### Other complexities

Mothers also discussed several challenges that were not part of the checklist. For example, families often cited a complicated and confusing registration process: *“the registration in the hospital took so long to process, it was so complicated”*; *“what made us stressed was the process, we had to prepare this letter and that letter and so many [other] letters and we didn’t know what we had to prepare”*. In 2 cases, mothers decided to change hospitals due to lack of care at the point of admission: *“We were at a different hospital before but the doctor didn’t check on us at all”*; *“at first, we went to the hospital but then they said it is not possible to stay there [due to crowding] … and then I made a decision with my husband to go to the clinic, and they [hospital staff] said that I can’t give birth there, I don’t know the reason why … and then we made decision to go to another hospital”*. Having to leave the hospital prior to admission increased women’s feelings of invisibility and lack of faith in the healthcare system.

Women often cited the need for privacy and the desire to be comfortable. In several instances they reported feelings of shame and trauma during childbirth, because their bodies were frequently exposed to numerous health workers, patients, and their families in the labour ward. Overcrowding in the rooms, in some cases due to the presence of medical students, overwhelmed women and contributed further to their feelings of shame and trauma: *“When the other patient is about to give birth, the male medical students or doctors also come in”*; *“as I’m giving birth for the first time, I can hear the one beside me screaming so loudly and it’s kind of influencing me”*. In particular, the presence of staff members induced feelings of lack of consideration for their privacy and dignity: *“there were ten students who wanted to observe and I rejected them”*; *“when the baby was born, they took it and took lots of picture of the baby and I didn’t like it”*; *“they told me to breastfeed the baby … and they took pictures at that time”*. Attempting to maintain bodily sanctity and modesty during childbirth left many women feeling ashamed. While overcrowding and the presence of medical students varied between facilities, lack of privacy and undignified treatment were consistent themes among mothers.

Almost half of the mothers that we interviewed (11 out of 28, 8 of which gave birth through C-section) indicated mistrust of the healthcare system. Many women who had caesareans expressed feelings of being ignored or coercion resulting in a lack of choice. It should be noted that in all of the cases in our sample, C-sections were carried out due to birth complications; however, due to the lack of communication, the mothers and their companions remained suspicious: *“When we suggested to still wait for the normal birth, it seemed like that they were angry”*; *“I thought if I keep insisting for the normal birth, [then] they will blame me if something goes wrong with the baby … we were worried that if we make too many complaints, then the next service will not be good.”*

## Discussion

Including the voices of women to hear and understand their needs when seeking maternal care is central to establishing a checklist that empowers both health workers and the women and families that they serve. Providing space in the checklist that accounts for the visceral experiences of birth has the ability to create a more holistic checklist that acknowledges the personhood of women and the cultural practices of pregnancy and childbirth in Aceh. Small adaptations have the capacity to improve communication between patients and providers and encourage conversations about dignified care. Our findings are similar to observations from other developing countries [33] that stress the importance of effective communication to achieve a good quality care. A prominent theme throughout our findings was the lack of communication between health providers and the mothers/their birth companions. As a result, mothers’ experiences were incongruous to their expectations, leaving most of them disappointed with the care they received. In particular, informing mothers about danger signs to look out for pre and post birth, support for early initiation of breastfeed, and adequate information on choices for family planning were areas that lacked effective communication. Implementing the SCC with points that emphasize the need for communication might help to ensure that informing mothers becomes integral to standard good practice across all maternal healthcare providers.

It should be noted that implementing some of the checklist items may overburden both the healthcare facilities and the women themselves. For example, the recommendation that women stay in the facility for 24 h after giving birth may not be possible due to lack of space and resources. Additionally, mothers often had other children and responsibilities that prompted them to leave the health facility earlier than 24 h after giving birth. As mothers were often accompanied by their family members, it was often not possible for them to stay long when there were no obvious danger signs.

Lack of care at the point of admission and complicated processes were other common theme noted by mothers. Fortunately, in our study such complications did not cause any serious negative outcomes. However, other studies indicate that this is a key point at which not receiving essential care, lack of clear guidance on rights to free insurance, and lack of adequate attention paid by the facility staff might lead into losing mother and/or baby [17].

Evidence indicates that maintaining privacy and dignity is key to mothers’ satisfaction with the provision of maternal care, a point that has also been recognised by the WHO [34]. However, particularly in low- and middle-income countries, undignified treatment remains an issue that affects health outcomes and contributes to women being reluctant to seek institutionalized maternal care [35] [36]. Effective communication with mothers and their families, care with respect and dignity, access to social and emotional support, and access to the necessary physical resources for good QoC are cross-cutting areas of the framework that can be adapted to the SCC [16]. While the SCC’s point-by-point structure helps to identify areas for improvement, it needs to be integrated to meet the wider aspects and challenges of providing quality care. We should also note that some of the steps of the SCC are tangled with cultural complexities that require a deeper understanding in order to confront the realities of how women navigate structural cultural systems.

### Strengths and limitations

Adopting a qualitative approach prior to the implementation of the SCC allowed us to gain a deeper insight into the lived experiences of mothers and how they can be translated into the adaptation of the checklist. Efforts to adapt the checklist to meet local needs have yet to include the experiences of women engaging with the health facilities; continuing biomedical births [37]. Our study aimed to bring personhood into the checklist while recognizing the multifaceted dimensions of QoC. We found studies that focus on incorporating women’s voice in the adaptation of the SCC to be particularly scarce. However, we do not claim that our findings are applicable to all mothers or throughout other regions. It should also be noted that these results are based on mothers’ recollections of their birth experiences and thus, requires them to conjure thoughts from an often chaotic event. Our study design aimed to minimize recall error by interviewing mothers who recently gave birth and unpacking their experience in chronological order to assist in prompting their memory. Additionally, it is highly likely that their perceptions of care contrast to those of the healthcare providers, as both are balancing different sets of tasks and challenges; namely giving birth in the face of institutional barriers and limitations. Future qualitative studies exploring the implementation of the SCC from the perspectives of healthcare providers, such as midwives and administrative staff, would be beneficial to gain a deeper understanding of the structural environment in maternity wards.

While our qualitative approach helped us to reduce the likelihood of social desirability bias, the presence of other family members during the interviews imposed complexities. It is possible that presence of family members influenced the mother’s responses; however, it is equally possible that it provided a sense of support and encouragement, helping mothers to remember moments of their experience.

Our sample included two cases of new born mortality. Although such cases are uncommon, we believe that excluding these cases would lead into missing valuable information. Lastly, a limitation of this study is the high number of women who gave birth through caesarean. This number is higher than figures for Aceh province of 22% in 2017 [28]. However, this high number can be attributed to the facility-based setting in which our study took place, as most women who went there to give birth were experiencing a complication or adverse event. In this route, further research exploring the feasibility of introducing the SCC to village midwives is needed.

## Conclusion

Our findings highlight the need to actively listen to and include the experiences of women in the adaptation and implementation of the checklist. Efforts to implement interventions targeted at women’s care need to engage with mothers to better contextualise the challenges experienced at each health facility. The findings of our study indicate that implementing the SCC has the potential to improve the quality of maternal care and overall birth experience. Specifically, emphasizing the communication of danger signs, encouraging breastfeeding, receiving information regarding family planning, and ensuring that women are aware of how to seek follow up care, can create more empowered patients. This work serves as an illustration of how a more holistic understanding of the lived experiences of women and the dynamics of their interactions with health facilities, providers and their birth companions can complement the implementation of the checklist.

## Supplementary information

**Additional file 1:** WHO Safe Childbirth Checklist.

**Additional file 2: Table S1.** Maternal care indicators among women aged 15-49 in Aceh province, Indonesia DHS 2017 (28). **Table S2.** Provision of healthcare in Aceh province 2017 (29). **Table S3.** Summary characteristics of facilities included in the main study population and interview sample stratified by facility type (annual figures for August 2015 to July 2016).

**Additional file 3:** Interview Guidance.

## Data Availability

Data presented in this study is available upon reasonable request through the corresponding author.

## Abbreviations

BEmONC: Basic Emergency Obstetric and Newborn Care
CEmONC: Comprehensive Emergency Obstetric and Newborn Care
MDG: Millennium Development Goal
MMR: Maternal Mortality Ratio
QoC: Quality of Care
RCT: Randomized control trial
SCC: Safe Childbirth Checklist
SDG: Sustainable Development Goal
WHO: World Health Organization
