# Supplementary material for: Mothers’ experiences of quality of care and potential benefits of implementing the WHO safe childbirth checklist: a case study of Aceh Indonesia
Source: BMC Pregnancy Childbirth. 2019 Dec 3;19:461. doi: 10.1186/s12884-019-2625-8 (PMC6891962; doi:10.1186/s12884-019-2625-8)
Supplement: Supplementary file 2 — Additional file 2: Table S1. Maternal care indicators among women aged 15-49 in Aceh province, Indonesia DHS 2017 (28). Table S2. Provision of healthcare in Aceh province 2017 (29). Table S3. Summary characteristics of facilities included in the main study population and interview sample stratified by facility type (annual figures for August 2015 to July 2016). [file 12884_2019_2625_MOESM2_ESM.pdf]

Additional file 2

Table S1. Maternal care indicators among women aged 15-49 in Aceh province, Indonesia DHS 2017 (28).

Indicator	% Rate
Receiving antenatal care from a skilled provider*	96.5
Receiving antenatal care from a skilled provider, at least four visits*	63.3
Had complications associated with the pregnancy*	18.2
Delivered in a health facility*	78.7
Delivered by a skilled provider*	84.5
Delivery by C-section	21.6
Neonatal mortality**	19
Post-neonatal mortality**	11
Had postnatal check during the first 2 days after birth*	85.2
Ever breastfed last-born children born in the past 2 years*	92.7
Current use of modern contraception (all and currently married women)	41.4

*Had a live birth in the 5 years preceding the survey.

**Per 1,000 live births for the 10-year period preceding the survey.

Table S2. Provision of healthcare in Aceh province 2017 (29).

Indicator	Value
Ratio of hospital beds per 1,000 population	1.72
Percentage of community health centres with fair/excessive number of physicians	87.02
Percentage of community health centres with fair/excessive number of nurses	84.96
Percentage of community health centres with fair/excessive number of midwives*	95.87
Number of obstetric gynaecologists at hospitals per 1,000 population	0.03
Number of midwives at hospitals per 1,000 population	0.49

*An outpatient health center is required to employ at minimum four midwives, while an inpatient health center seven midwives.

Table S3. Summary characteristics of facilities included in the main study population and interview sample stratified by facility type (annual figures for August 2015 to July 2016).

	Study population* (*N* = 29)	Interview sample (*N* = 7)
Median number of obstetricians/gynecologists part/full-time		
Puskesmas	0	0
Public hospital	5	8
Private hospital	2	3
Median number of midwives part/full-time		
Puskesmas	16.5	37
Public hospital	17	23.5
Private hospital	10.5	13
Median number of total staff (part/full-time)		
Puskesmas	16.5	37
Public hospital	24	32.5
Private hospital	14	16
Median number of labor rooms		
Puskesmas	1	1
Public hospital	1	2
Private hospital	1	1
Median number of operation theaters		
Puskesmas	0	0
Public hospital	2	2.5
Private hospital	1	1
Median number of Bedsides for mothers		
Puskesmas	4	8
Public hospital	6	6
Private hospital	8.5	3.5
Median number of inward referrals		
Puskesmas	25	0
Public hospital	227	1023
Private hospital	118	1030
Median number of outward referrals		
Puskesmas	32.5	124
Public hospital	9	5..5
Private hospital	11	27.5
Median number of deliveries (all type)		
Puskesmas	43	127
Public hospital	741	988.5
Private hospital	349.5	940.5

Ratio of cesarean by total deliveries (%)		
Puskesmas	4.26	0
Public hospital	32.01	32.45
Private hospital	63.22	75.44
Ratio of maternal deaths by total deliveries (%)		
Puskesmas	0.12	0
Public hospital	0.38	0.15
Private hospital	0.11	0.06
Ratio of stillbirths by total deliveries (%)		
Puskesmas	1.34	0.79
Public hospital	1.35	1.47
Private hospital	0.81	0.74
Ratio of newborn deaths by total deliveries (%)		
Puskesmas	0.24	0
Public hospital	0.23	0.04
Private hospital	0.73	0.69

*The main RCT study also included a small number of private midwife clinics and sub-health centers (pustu) that were not included in the qualitative study due to their small proportion.
