# Supplementary material for: Mothers’ experiences of quality of care and potential benefits of implementing the WHO safe childbirth checklist: a case study of Aceh Indonesia
Source: BMC Pregnancy Childbirth. 2019 Dec 3;19:461. doi: 10.1186/s12884-019-2625-8 (PMC6891962; doi:10.1186/s12884-019-2625-8)
Supplement: Supplementary file 3 — Additional file 3: Interview Guidance. [file 12884_2019_2625_MOESM3_ESM.pdf]

Additional file 3: Interview Guidance

Location:<br>AB / B/ BA	Code:	Family present during interview:

Start by introducing ourselves and thanking them for their time and inviting us into their home.

Reason for the interview: we are part of a research team working with the WHOs Safe Childbirth Checklist. We would like to talk to you about your experiences during childbirth to learn about your perceptions and experiences with care during childbirth. We are hoping that your stories will help inform the checklist to improve the quality of care that mothers receive. There are no right or wrong answers.

**Go through the consent form: the talk will take about an hour, everything they say will be kept confidential and their names will be kept hidden. Ask to audio record the interview so we can listen to it again for transcription and data analysis. Check for questions.

Before we begin we just have some background questions for you:

Age	
Education	
Occupation	
No. Of Children	
Where did you give birth?	
Do you have BPJS?<br>What class?	

Please note that this is a semi-structured interviewed guide. Therefore, interviews did not follow the formalized list of questions below, but allowed for a discussion with the interviewee following the four pause points of the checklist.

Topic	Question	Probe	Notes
**On Arrival**	Did you have any concerns about going to the health facility?	• What were they?	
	Did you have any challenges accessing care and getting to the HF?	• Time, cost, travel, children, family, perceptions	
	Can you tell us about your experience when you first arrived at the HF?	• How long was the waiting time, when you first arrived until you got treated<br>• Did you feel comfortable	

		(going to) in a health facility?<br>• Was it clean?	
	Did someone from your family come with you	If the family is there you can ask them directly:<br>• Were the staff nice to them and sensitive to their needs?<br>• Were they informed about what was happening and when to seek help?	
	At any time during your birth, were you or your baby referred to another HF?	If YES:<br>• Can you tell us about this experience?<br>• Did they inform you why you need to be referred to other HF?<br>• Did you pay anything for this?	
**Just before pushing/or before caesarean)**	How frequently did the midwives/nurse/doctors come to check on you?	• When they came did they inform you about what was happening with you and your baby?	
	Did the HPs ask for your consent before they did everything?	• Ie. Before checking dilation, checking blood pressure, etc.<br>• Were procedures explained to you?<br>• Were you given the results?	

	Did the HF ask you to provide any supplies for your birth?	• i.e. Any medication? Bed sheets? etc.	
**During birth**	During your birth did you feel like you were in control?	• Were you listened to?<br>• Concerns taken seriously?<br>• Were you able to pick the position that you gave birth in?	
	In your opinion, was your privacy and modesty respected?	• Were you able to request a female staff?<br>• Were you draped before giving birth?<br>• Comfortable to keep your hijab on or take it off?	
**Soon After Birth**	After your birth, did the HP encourage you to breastfeed your baby and do skin-to-skin contact?		
**Before Discharge**	How long did you stay at the facility before you went home?	• Did you feel that this was long enough, or would you have liked to stay longer?	
	At any time during your care, before or after pregnancy, did anyone talk to you about family planning?	• What did they say?<br>• Do you think you were given enough information or would you have liked to know more?	

	Have you and your baby been getting follow-up care?	If they answer YES:<br>• Are there any challenges to accessing the care?<br>• Do you get information about the danger sign for you and the baby?<br>• Do you know where to go/ call to find help<br><br>If they answer NO:<br>• Why not, are there any challenges that you have in accessing the care?	
**Final Questions**	What does good care during childbirth mean to you?	• Probe for examples<br>• Trying to figure out if they feel like they received good care	
	Is there any way in which you feel that your birth could have been improved?	• Recommendation for the improvement of the HF?	
	Is there anything else about the care you and your baby received during your birth that you would like to tell us about?		
